# Association between metabolic syndrome and hearing loss: The mediating role of retinol – A cross-sectional analysis of NHANES 2007 to 2018 (excluding 2013–2014)

**DOI:** 10.1097/MD.0000000000049234

**Published:** 2026-06-05

**Authors:** Linwei Mao, Zhiyong Pan, Weiqun Hu, Huiting Dai, Xiufen Chen

**Affiliations:** a School of Clinical Medicine, Fujian Medical University, Fujian, PR China; b Department of Otolaryngology, Affiliated Hospital of Putian University, Putian, Fujian, PR China.

**Keywords:** hearing loss, metabolic syndrome, MetS, retinol, vitamin A

## Abstract

Emerging evidence suggests associations between metabolic syndrome (MetS) and hearing loss, though population heterogeneity remains understudied. Given the high susceptibility of the inner ear to oxidative damage, fat-soluble antioxidants such as vitamins A, E, and carotenoids may exert protective effects. This study aimed to examine the MetS–hearing loss association among the U.S. adults and assess the potential mediating roles of these antioxidants. This cross-sectional study analyzed data from the U.S. National Health and Nutrition Examination Survey (2007–2018, excluding 2013–2014). Weighted multivariable linear regression models were employed to assess the association between MetS and pure-tone average (PTA) thresholds. Hearing loss was defined as a PTA ≥ 20 decibel in the better ear. Subgroup analyses explored effect modification by demographics (age, sex, race, education, marital status, income) and exposures (noise, ear infection history). Sensitivity analyses, including complete-case analysis and stratification by survey cycle, were conducted to test robustness. Mediation effect models were applied to evaluate the potential intermediary roles of vitamin A, vitamin E, and carotenoids. The study included 8759 U.S. adults (mean age 49.2 years; 51.1% female; 37.2% non-Hispanic White). Socioeconomically, 56.8% had college-level education or above, 59.2% were married or living with a partner, and 38.3% were in the middle-income tier. Significant noise exposure was reported by 48.9%, and 25.5% had a history of ear infections. MetS prevalence was 35.2%. After full adjustment, MetS showed a significant association with higher PTA thresholds after covariate adjustment (β = 1.292 decibel, 95% confidence interval: 0.315–4.106, *P* < .001). Subgroup analyses revealed stronger associations in older adults, non-Hispanic White, middle-income groups, and those with ear infection history (all interaction *P* < .05). Mediation analysis identified retinol (vitamin A) as the only significant mediator among 13 fat-soluble antioxidants, demonstrating an indirect effect of β = −0.358 (95% confidence interval: −0.847 to −0.061, *P* *=* .020) with a mediation proportion of −13.3%. MetS is independently associated with hearing threshold elevation, with heterogeneity across subgroups. Retinol may play a crucial mediating role by modulating the inner ear oxidative–antioxidative balance. Prospective cohort studies and mechanistic investigations are needed to verify causality and elucidate underlying pathways, potentially informing novel strategies for preventing MetS-related hearing loss.

## 1. Introduction

Hearing loss, as one of the most prevalent sensory dysfunctions worldwide, has emerged as a growing public health challenge. According to the latest comprehensive estimates from the World Health Organization (WHO), approximately 1.5 billion people globally suffer from varying degrees of hearing loss, including over 430 million individuals with moderate to severe hearing loss. This figure is projected to exceed 2.5 billion by 2050 (WHO, 2021).^[1]^ In the United States, hearing loss affects a substantial proportion of the population: about 15% of adults (37.5 million) report some trouble hearing, and this prevalence rate has remained relatively stable over the past 2 decades.^[2,3]^ Beyond impairing speech communication and social engagement, hearing loss is closely associated with a 30% increased prevalence of subjective cognitive decline and a 35% elevated risk of depression; moreover, it interacts with depression to jointly exacerbate cognitive decline, imposing a substantial burden on both families and society.^[4–6]^ Although preventive public health strategies and lifelong clinical interventions can mitigate hearing loss, current public health practices still face significant challenges in prevention due to the complexity and diversity of risk factors (e.g., aging, noise exposure, chronic diseases) and the incomplete understanding of their interactions.^[7]^

Hearing loss is a complex pathological process involving multiple factors, with oxidative stress being recognized as one of the key mechanisms. The cochlear tissue, rich in unsaturated fatty acids and possessing a vulnerable antioxidant system, is particularly susceptible to oxidative damage. Excessive accumulation of reactive oxygen species (ROS) can trigger lipid peroxidation, DNA damage, and hair cell apoptosis, ultimately leading to auditory dysfunction.^[8,9]^ The body’s endogenous antioxidant system plays a crucial protective role in maintaining redox homeostasis. Exogenous antioxidants such as vitamin A, vitamin E, and carotenoids contribute to this protection by scavenging ROS, upregulating the activity of antioxidant enzymes like superoxide dismutase, and preserving cellular redox balance.^[10–13]^ Among these, retinol (vitamin A) and its active metabolite, retinoic acid, play particularly important roles in maintaining inner ear homeostasis. The retinol/retinoic acid signaling axis is critical for zonal patterning of vestibular organs through synthesis and autoregulatory mechanisms during inner ear morphogenesis.^[14]^ Moreover, recent advances have revealed that this signaling axis governs dual functions in cochlear organogenesis, coordinating both progenitor cell proliferation and differentiation through context-dependent transcriptional regulation.^[15]^ Studies have demonstrated that these compounds can inhibit hair cell apoptosis and play a significant role in maintaining hair cell function.^[16]^ Further in vitro experiments have shown that carotenoids can directly bind to cochlear cell membranes and neutralize free radicals generated by lipid peroxidation, thereby protecting hair cells from oxidative damage.^[12]^ Oral supplementation of vitamin A and vitamin E has been shown to mitigate noise-induced hearing loss by promoting the survival of sensory hair cells.^[17]^ This cochlear protective mechanism involves the regulation of antioxidant enzymes and apoptosis-related proteins in the cochlea.^[12,18]^ These findings highlight the multifaceted role of vitamin A in auditory system integrity. Recent population-based studies have further substantiated the protective role of dietary antioxidants against hearing impairment: Min et al reported an L-shaped association between the Composite Dietary Antioxidant Index and hearing loss, while Fu et al demonstrated an inverted-U relationship between dietary inflammatory potential and hearing outcomes.^[19,20]^ However, the precise roles of vitamin A, vitamin E, and carotenoids in the progression of hearing loss remain to be fully elucidated.

Metabolic syndrome (MetS), a complex cluster of metabolic disorders encompassing obesity, hypertension, dysglycemia, and dyslipidemia, represents a constellation of cardiovascular risk factors. With a global prevalence exceeding 25% and an increasing trend among younger populations,^[21]^ its burden in the United States is substantial and growing. Recent analyses of nationally representative data indicate that the weighted prevalence of MetS among U.S. adults reached 38.7% in recent cycles, with a significant upward trajectory observed over past decades – from 28.23% during 1999 to 2000 to 37.09% by 2017 to 2018.^[22,23]^ Given this substantial and growing burden of MetS, its potential association with hearing loss has garnered increasing attention. Emerging evidence suggests that MetS may be a potential risk factor for hearing loss.^[24–26]^ Pathophysiologically, components of MetS may contribute to hearing loss by compromising cochlear microvascular integrity.^[27,28]^ Large-scale studies indicate that MetS is associated with increased odds of sensorineural hearing loss, with reported estimates ranging from 11% to 38% higher odds compared to those without MetS.^[24,25]^ This risk shows a dose-dependent relationship with the number of MetS components, where hearing loss prevalence rises from 7.9% (no components) to 16.3% (all 5 components).^[26]^ Additionally, analyses focusing on individual components have found that central obesity (OR = 1.07), hyperglycemia (OR = 1.12), and low high-density lipoprotein cholesterol (HDL-C) levels (OR = 1.21) are independently associated with increased risk.^[25]^ Collectively, preliminary studies indicate statistical links between MetS and hearing loss, yet population heterogeneity remains uncharacterized.

MetS may interfere with the metabolism and bioavailability of fat-soluble antioxidants through multiple pathways. First, the chronic low-grade inflammation associated with MetS can affect hepatic function, potentially disrupting the synthesis of transport proteins such as retinol-binding protein 4 and thereby impairing vitamin A delivery to target tissues. Concurrently,^[29]^ inflammatory responses may deplete antioxidant reserves including vitamin E and carotenoids. Second, hyperglycemia and lipid peroxidation – core components of MetS – can increase ROS generation, accelerating antioxidant consumption and reducing total antioxidant capacity^[30]^. Additionally, adipose tissue in obese states serves as a crucial reservoir for fat-soluble vitamins^[31]^; its dysfunction may impair the storage and release of retinol, vitamin E, and carotenoids – epidemiological studies have confirmed significant associations between obesity and lower serum concentrations of carotenoids and vitamin E.^[32]^ Notably, emerging evidence has identified retinol metabolism signaling as a critical pathway linking metabolic dysregulation to systemic homeostasis. In obese mice, retinol metabolism signaling was shown to mediate microbiota-regulated fat deposition, indicating that MetS-associated dysbiosis can directly disrupt retinol metabolic flux through the gut–liver axis.^[33]^ This suggests that beyond serving as an antioxidant, retinol metabolism itself is actively involved in the pathophysiology of MetS. Thus, MetS may compromise inner ear antioxidant defense by altering the metabolic stability of retinol and other fat-soluble antioxidants, potentially contributing to the development of hearing impairment. Given the central role of retinol in cochlear homeostasis and its susceptibility to metabolic dysregulation, it represents a particularly compelling candidate to mediate the relationship between MetS and hearing loss.

The National Health and Nutrition Examination Survey (NHANES) is uniquely positioned to address these research gaps. It provides nationally representative data, concurrent assessments of hearing function and MetS components, measurements of antioxidant levels, comprehensive covariates for robust statistical adjustment, and a sample size sufficient for detailed mediation and subgroup analyses. Leveraging the NHANES database, this study aims to examine the MetS–hearing threshold relationship, investigate potential mediating roles of vitamins A, E, and carotenoids in this association, and provide observational evidence supporting shared pathological pathways between these disorders.

## 2. Materials and methods

### 2.1. Study population

This study utilized data from the NHANES, a biennial survey that employs a complex, multistage probability sampling design to collect representative health and nutritional information from the non-institutionalized U.S. population across all 50 states and the District of Columbia. The database includes detailed demographic characteristics, lifestyle factors, and various health metrics.

The NHANES employs a stratified, multistage probability sampling design to ensure a nationally representative sample of the non-institutionalized civilian U.S. population. The sampling process involves 4 primary stages: (1) primary sampling units: selection of counties or small groups of contiguous counties. (2) Segments within primary sampling units: selection of city blocks or other geographically defined areas. (3) Households within segments: random selection of dwelling units. (4) Individuals within households: finally, specific individuals are sampled with differential probabilities across demographic subgroups (e.g., oversampling of older adults, racial/ethnic minorities, and low-income persons) to improve the precision of estimates for these groups. This complex design necessitates the application of sample weights, clustering, and stratification variables in all analyses to obtain unbiased estimates and correct standard errors.

To examine the association between hearing loss and MetS, we analyzed data from 4 NHANES cycles (2009–2018). The 2013 to 2014 cycle was excluded because the audiometry examination component was not administered during that survey period, as per the NHANES survey design. The hearing examination is a specialized module that is not fielded in every biennial cycle due to evolving public health priorities and resource allocation. The inclusion criteria for the present analyses were: (1) aged 20 years or older; (2) availability of complete data for the diagnosis of MetS based on National Cholesterol Education Program Adult Treatment Panel III criteria; and (3) completion of the audiometric examination with available pure-tone average (PTA) thresholds. Participants not meeting all 3 criteria were excluded from the primary complete-case analysis.

### 2.2. Definition of MetS

MetS was diagnosed according to the National Cholesterol Education Program Adult Treatment Panel III guidelines.^[12]^ Participants were identified as having MetS if they met 3 or more of the following criteria: (1) central obesity (waist circumference ≥102 cm for men or ≥88 cm for women), (2) hypertriglyceridemia (≥150 mg/dL or current use of triglyceride-lowering agents), (3) low HDL-C (<40 mg/dL for men or <50 mg/dL for women), (4) hypertension (≥130/85 mm Hg or current antihypertensive medication use), and (5) hyperglycemia (fasting glucose ≥100 mg/dL or current antihyperglycemic medication use).^[18,19]^

### 2.3. Audiometric assessment

Pure-tone air-conduction audiometry was conducted, covering the main frequency range of human hearing (500, 1000, 2000, and 4000 Hz), following protocols in the Audiometry Procedures Manual published by the National Center for Health Statistics. Air-conduction thresholds (decibel hearing level) were measured using professionally calibrated equipment. The PTA was calculated as the arithmetic mean of thresholds at these 4 frequencies, with the ear demonstrating the lower PTA (better hearing acuity) serving as the representative value for each participant.^[20]^ Consistent with the WHO World Report on Hearing,^[1]^ hearing loss was defined as PTA ≥ 20 decibel (dB) in the better ear.

### 2.4. Covariates

Sociodemographic data, including age, sex, race, education level, marital status, and the ratio of family income to poverty (family PIR), were collected through self-reported interviews. For race/ethnicity, Mexican Americans, other Hispanics, and individuals of other racial backgrounds were combined into a single “other” category. education level was categorized as high school graduate or less versus college or higher. Marital status was classified as married/living with partner, never married, and widowed/divorced/separated. Family PIR was stratified into 3 tiers: ≤130%, >130 to 350%, and >350%.

Additionally, hearing loss-related risk factors (noise exposure and history of ear infections) were included as covariates. Noise exposure (yes/no) was defined based on affirmative responses to workplace, leisure-time, or firearm-related noise exposure, coupled with infrequent use of hearing protection (no more than half of the time when exposed to loud noise). A history of ear infections was defined as a self-reported “yes” to either “Ever had tube placed in ear” or “Ever had 3 or more ear infections.”

### 2.5. Statistical analysis

Participants’ characteristics were summarized using descriptive statistics: continuous variables were presented as mean ± standard deviation, and categorical variables as frequency (percentage). The normality of continuous variables was initially assessed using visual inspection (histograms and Q–Q plots) and the Shapiro–Wilk test. Given the large sample size, which renders formal normality tests overly sensitive, we primarily relied on the evaluation of skewness and kurtosis. Variables with absolute skewness >3 and/or absolute kurtosis >10 were considered to have substantial deviation from normality. For comparisons of hearing thresholds, continuous variables were analyzed using the Mann–Whitney *U* test or Student *t* test based on their distribution, while categorical variables were assessed with the chi-square test. Given the complex sampling design of NHANES, sampling weights were incorporated into all analyses. Statistical analyses were performed using R software version 4.5.1, with a 2-tailed *P* < .05 considered statistically significant.

The association between MetS and PTA was examined using multivariable linear regression models with 3 sequential adjustments: Model 1 (demographic-adjusted) adjusted for age, sex, and race; Model 2 (socioeconomic-adjusted) additionally adjusted for education level, marital status, and family PIR; Model 3 (fully adjusted) further incorporated noise exposure and history of ear infections as important confounding factors.

Exploratory stratified analyses were conducted to evaluate the association between MetS and pure-tone thresholds across population subgroups. All stratified models maintained the same full adjustment as in the primary Model 3, including age, sex, race, education, marital status, family PIR, noise exposure, and history of ear infections, with only the stratification variable itself excluded. Multiplicative interaction tests were employed to quantitatively assess potential interaction effects between covariates and MetS. To specifically explore age-related differences in the MetS–hearing threshold association, participants were stratified into higher age (≥50 years) and lower-age (<50 years) groups based on the average age. Moreover, to enhance the robustness of the findings, we conducted sensitivity analyses restricted to participants with complete covariate data, excluding those with missing values.

In the final analytical sample, partial covariate data were missing. We characterized the missingness mechanism and evaluated its potential impact on population representativeness. To address potential bias from complete-case analysis and under the assumption that data were missing at random, we implemented multiple imputation by chained equations (MICE) using the mice package in R, creating 5 imputed datasets. All covariates listed in Section 2.4, along with the exposure (MetS) and outcome (PTA), were included in the imputation model. Results from analyses based on the imputed datasets were consistent with the primary complete-case findings presented in the main text. For transparency, the main results are reported based on the complete-case analysis, with sensitivity analyses performed to verify robustness (see Section 3.6). To further assess sample size adequacy, we performed a post hoc power analysis based on Model 3 (fully adjusted), which demonstrated >99% power to detect the observed association (β = 1.292 dB) at α = 0.05 (2-tailed), confirming sufficient sample size and minimal Type II error risk for the primary outcome.

The analyzed fat-soluble antioxidants comprised 13 compounds from 3 categories: (1) vitamin A compounds: retinol, retinyl palmitate, and retinyl stearate; (2) vitamin E isomers: γ-tocopherol and α-tocopherol; (3) carotenoids: α-carotene,trans-β-carotene, cis-β-carotene, lutein/zeaxanthin, trans-lycopene, total lycopene, α-cryptoxanthin, and β-cryptoxanthin. Mediation analysis was conducted using the “mediation” package in R to examine the potential mediating roles of 13 fat-soluble antioxidants (specifically, vitamin A compounds, vitamin E isomers, and carotenoids; the complete list is provided in Table 4) in the MetS–PTA association. To properly account for the complex sampling design of NHANES (stratification, clustering, and sampling weights), we fitted the mediator and outcome models using survey-weighted generalized linear models with the survey package. Mediation effects were then assessed via nonparametric bootstrap with 5000 resamples using the mediation package, incorporating the survey weights to obtain bias-corrected 95% confidence intervals (CIs) for the indirect effects. A mediation effect was considered statistically significant if the 95% CI excluded 0. All models were adjusted for sex, age, race, education level, marital status, family PIR, noise exposure, and history of ear infections.

## 3. Results

### 3.1. Participant selection

This study utilized data from 4 NHANES cycles (2009–2018, excluding 2013–2014). From an initial pool of 47,601 participants, we sequentially excluded those aged <20 years (n = 19,777), those with incomplete MetS diagnostic data (n = 2805), and those missing audiometry results (n = 16,260). The final analytical sample comprised 8759 eligible adults.

To evaluate potential selection bias, we compared baseline characteristics between included and excluded participants who were aged ≥20 years (Table S1, Supplemental Digital Content 1). The standardized mean differences (SMDs) for all covariates were small (│SMD│≤0.16), with most below the conventional threshold of 0.1.

### 3.2. Baseline characteristics

The baseline characteristics of the 8759 participants are presented in Table 1, stratified by hearing status. The mean age was 49.16 (± 17.73) years, with 51.11% being female. Regarding race/ethnicity, 37.21% were non-Hispanic White, 23.03% non-Hispanic Black, and 39.76% belonged to other groups (specifically, 13.70% Mexican American, 10.89% other Hispanic, and 15.17% other race). More than half (56.81%) had a college education or above, and 59.20% were married or living with a partner. The most common family PIR category was 1.3 to 3.5 (38.33%). Nearly half (48.90%) reported significant noise exposure, 25.48% had a history of ear infections, and 35.24% were diagnosed with MetS.

**Table 1:** Baseline characteristics of the U.S. adult study population (NHANES 2009–2018, excluding 2013–2014), stratified by hearing status.

Total	All (n = 8759)	Hearing loss (n = 1985)	Normal hearing (n = 6774)	*P* value
Sociodemographic Characteristics				
Age, mean ± SD	49.16 ± 17.73	67.82 ± 25.27	43.69 ± 26.23	<.001
Sex, n (%)				<.001
Female	4477 (51.11)	856 (43.12)	3621 (53.45)	
Male	4282 (48.88)	1129 (56.88)	3153 (46.55)	
Race, n (%)				<.001
Non-Hispanic White	3259 (37.21)	1056 (53.2)	2203 (32.52)	
Non-Hispanic Black	2017 (23.03)	317 (15.97)	1700 (25.1)	
Others	3483 (39.76)	612 (30.83)	2871 (42.38)	
Others (Mexican American)	1200 (13.70)	245 (12.34)	955 (14.10)	
Others (other Hispanic)	954 (10.89)	180 (9.07)	774 (11.43)	
Others (other race)	1329 (15.17)	187 (9.42)	1142 (16.86)	
Education level, n (%)				<.001
High school graduate or less	3783 (43.19)	1105 (55.67)	2678 (39.53)	
College or above	4976 (56.81)	880 (44.33)	4096 (60.47)	
Marital status, n (%)				<.001
Married/living with a partner	5185 (59.20)	1155 (58.19)	4030 (59.49)	
Never married	1732 (19.77)	139 (7.00)	1593 (23.52)	
Divorced/separated/widowed	1842 (21.03)	691 (34.81)	1151 (16.99)	
Family PIR, n (%)				<.001
≤1.3	2750 (31.4)	586 (29.52)	2164 (31.95)	
1.3–3.5	3357 (38.33)	860 (43.32)	2497 (36.86)	
>3.5	2652 (30.28)	539 (27.15)	2113 (31.19)	
Hearing-related risk factors				
Noise exposure, n (%)				<.001
Yes	4282 (48.90)	1086 (54.71)	3196 (47.19)	
No	4475 (51.10)	899 (45.29)	3576 (52.81)	
History of ear infections, n (%)				<.001
Yes	1933 (25.48)	337 (30.17)	1596 (24.67)	
No	5653 (74.52)	780 (69.83)	4873 (75.33)	
Metabolic syndrome, n (%)				<.001
Yes	3087 (35.24)	1146 (57.73)	1941 (28.65)	
No	5672 (64.76)	839 (42.27)	4833 (71.35)	

PIR, poverty–income ratio.

Hearing loss (PTA ≥ 20 dB in the better ear) was present in 1985 participants, accounting for 22.66% of the study population. Significant differences between groups (all *P* < .001): compared to the normal hearing group, participants with hearing loss were significantly older (67.82 vs 43.69 years) and more likely to be male (56.88% vs 46.55%). Racial distribution also differed markedly, with a higher proportion of non-Hispanic White individuals in the hearing loss group (53.20% vs 32.52%) and correspondingly lower proportions across all other racial/ethnic subgroups. The hearing loss group had a lower prevalence of higher education (44.33% vs 60.47% college or above), a different marital status profile (e.g., higher rates of being divorced/separated/widowed: 34.81% vs 16.99%), and a higher prevalence of MetS (57.73% vs 28.65%). A history of ear infections was also more common in the hearing loss group (30.17% vs 24.67%).

Despite overall statistical significance for some categorical variables, the absolute differences in the proportions of participants who were married or living with a partner (58.19% in HL vs 59.49% in NH) and those in the lowest family income tier (PIR ≤ 1.3) (29.52% vs 31.95%) were minimal. The distributions of sex within the normal hearing group and the specific composition of the “other races” category followed similar patterns relative to the total population structure in both groups.

### 3.3. Characteristics of participants by MetS status

The prevalence of individual MetS components in the total population and by MetS status is detailed in Table S2, Supplemental Digital Content 2. Among participants diagnosed with MetS (n = 3087), the most prevalent component was central obesity (92.32%), followed by low HDL-C (83.90%), hypertension (84.65%), hypertriglyceridemia (74.34%), and hyperglycemia (72.85%). In contrast, the prevalence of these components was substantially lower in the non-MetS group (n = 5672), ranging from 14.84% (hyperglycemia) to 51.64% (central obesity).

The characteristics of participants stratified by MetS status are shown in Table S3, Supplemental Digital Content 3. Of the 8759 participants, 3087 (35.24%) had MetS. The prevalence of hearing loss was more than twofold higher in the MetS group compared to the non-MetS group (37.12% vs 14.79%). Participants with MetS were significantly older and more likely to be female (both *P* < .001). They also had a lower proportion of college-educated individuals and a markedly different marital status profile, characterized by a higher rate of being divorced/separated/widowed and a lower rate of never being married (all *P* < .001). A history of ear infections was more common in the MetS group (*P* < .001).

Notably, despite statistical significance for family income (PIR, *P* < .001), the actual proportions in the lowest income tier (PIR ≤ 1.3) were similar between groups (32.30% in MetS vs 30.91%). The proportion of married individuals was also nearly identical (58.73% vs 59.45%). Other demographic factors, including the specific composition of racial subgroups and noise exposure, showed comparable distributions.

### 3.4. Association between MetS and hearing loss

As shown in Table 2, weighted multiple linear regression analyses revealed an association between MetS and hearing loss. In Model 1, which was adjusted for demographic factors such as sex, age, and race, MetS was significantly associated with a higher PTA (β = 1.750, 95% CI: 0.343–5.105, *P* < .001). This association remained significant after further adjustment for additional demographic factors including educational level, marital status, and family PIR (Model 2) (β = 1.411, 95% CI: 0.321–4.397, *P *< .001). In the fully adjusted model (Model 3), MetS was positively associated with hearing loss (β = 1.292, 95% CI: 0.315–4.106, *P* < .001).

**Table 2:** Association between metabolic syndrome (MetS) and pure-tone average (PTA) hearing thresholds in U.S. adults (NHANES 2009–2018, excluding 2013 to 2014).

	Model 1 (demographic-adjusted)		Model (socioeconomic-adjusted)		Model 3 (fully adjusted)	
	β (95% CI)	*P* value	β (95% CI)	*P* value	β (95% CI)	*P* value
Presence of MetS	1.750 (0.343–5.105)	<.001	1.411 (0.321–4.397)	<.001	1.292 (0.315–4.106)	<.001

Model 1: Adjusted for age, sex, and race.

Model 2: Combination of model 1 and education level, marital status, and family poverty–income ratio (PIR).

Model 3: Combination of model 2 and noise exposure and history of ear infections.

### 3.5. Subgroup analysis

**Figure 1.**
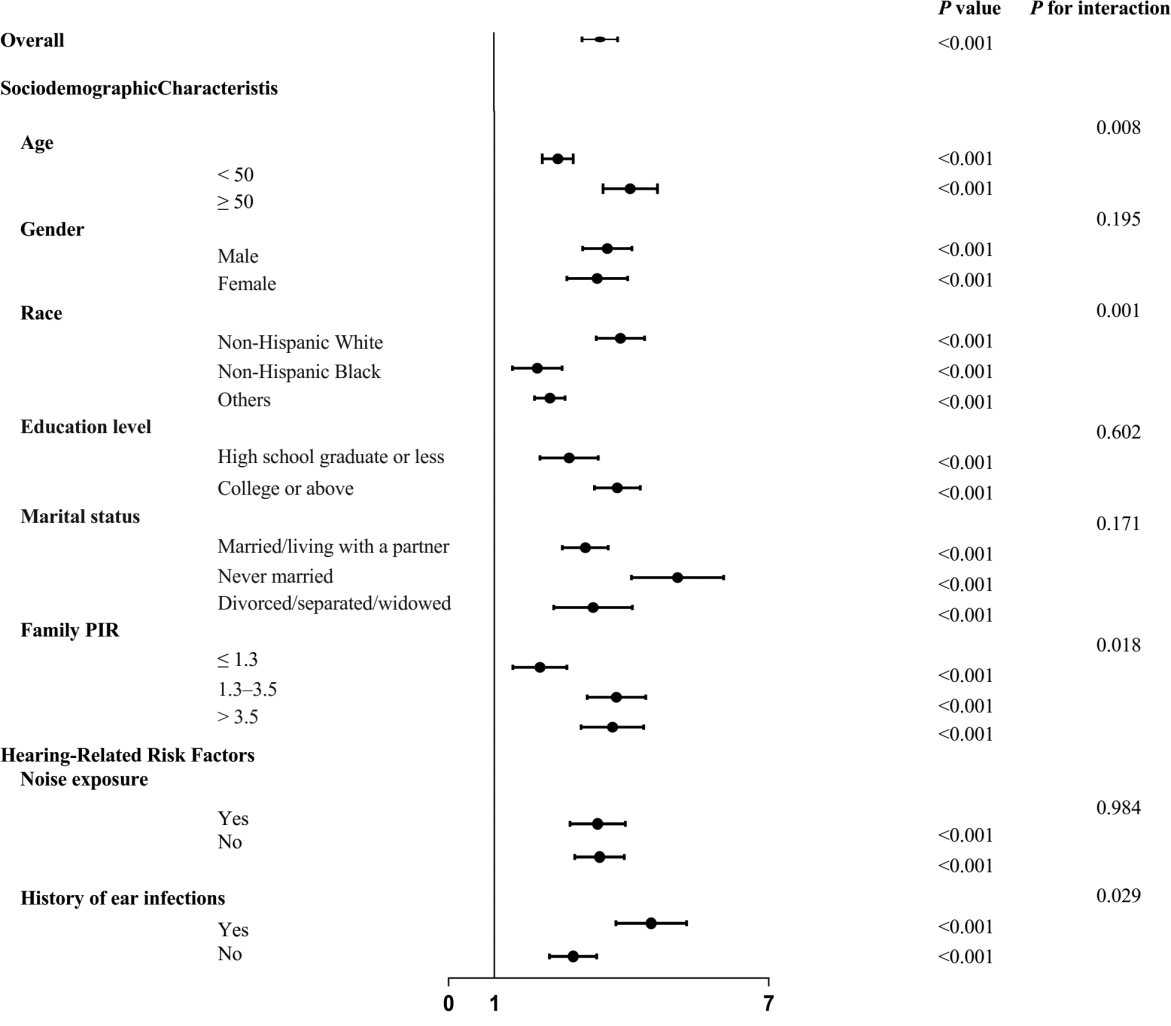
Forest plot of subgroup analyses for the association between metabolic syndrome (MetS) and pure-tone average (PTA) hearing thresholds among U.S. adults (NHANES 2009–2018, excluding 2013 to 2014). The forest plot presents weighted multivariable linear regression coefficients (β, in decibels [dB]) and 95% confidence intervals (CIs) for the association between MetS and better ear PTA, stratified by baseline covariates. All subgroup-specific models (points and horizontal lines) were fully adjusted for the same covariates as in the primary analysis (Model 3: age, sex, race, education, marital status, family PIR, noise exposure, and history of ear infections), except for the stratification variable itself. The diamond at the bottom represents the overall, fully adjusted association for the total study population (β = 1.292 dB, 95% CI: 0.315–4.106). Key findings indicate significant effect modification (interaction *P* < .05) in the following subgroups: age (≥50 vs <50 years), race (non-Hispanic White vs others), family PIR (middle-income vs low-income), and history of ear infections (yes vs no). The dashed vertical line at 0 indicates no association. CI, confidence interval; MetS, metabolic syndrome; PIR, poverty–income ratio; PTA, pure-tone average.

The association between MetS and PTA across different subgroups is presented in Figure 1. All subgroup models were adjusted for all covariates, and MetS was significantly positively associated with elevated PTA thresholds in all subgroups (all *P* < .001). Subgroups were stratified by demographic characteristics (age, sex, race, education, marital status, familyPIR) and exposure factors (noise exposure, history of ear infections).

Notably, interaction analysis revealed significantly stronger associations between MetS and PTA in the following subgroups (interaction *P* < .05): higher age (≥50 years vs <50 years; interaction *P =* .008), non-Hispanic White individuals (vs other racial/ethnic groups; interaction *P =* .001), middle-income individuals (vs low-income individuals; interaction *P =* .018), individuals with a history of ear infections (vs those without; interaction *P =* .029).

### 3.6. Sensitivity analysis

Complete-case sensitivity analysis was performed by excluding all participants with missing covariates across 4 survey cycles, with weighted multivariable linear regression methods consistent with the primary analysis to examine the MetS–PTA association. Detailed results of the complete-case sensitivity analysis are presented in Table 3, and the effect estimates and trends were consistent with the primary analysis.

**Table 3:** Sensitivity analysis of the association between metabolic syndrome (MetS) and pure-tone average (PTA) using a complete-case approach (NHANES 2009–2018, excluding 2013–2014).

	Model1 (demographic-adjusted)		Model (socioeconomic-adjusted)		Model 3 (fully adjusted)	
	β (95% CI)	*P* value	β (95% CI)	*P* value	β (95% CI)	*P* value
Presence of MetS	2.106 (0.370–5.698)	<.001	1.677 (0.340–4.932)	<.001	1.497 (0.331–4.523)	<.001

Model 1: Adjusted for age, sex, and race.

Model 2: Combination of model 1 and education level, marital status, and family poverty–income ratio (PIR).

Model 3: Combination of model 2 and noise exposure and history of ear infections.

Survey cycle-stratified sensitivity analysis was conducted to assess the temporal consistency of the MetS–PTA association, with results summarized in Table S4, Supplemental Digital Content 4. A significant positive association between MetS and PTA was observed in the 2011 to 2012 and 2015 to 2016 cycles (participants aged 20–69 years). In the 2017 to 2018 cycle (participants ≥ 70 years), the point estimate of the association was the highest among all cycles (β = 3.19), with borderline statistical significance (*P* = .059). No statistically significant association between MetS and PTA was found in the 2009 to 2010 cycle (participants ≥ 70 years).

### 3.7. Mediating role of retinol

Serum concentrations of multiple fat-soluble antioxidants differed significantly between the non-MetS and MetS groups (Table 4). Of the 13 antioxidants examined, 8 showed statistically significant associations with MetS status (*P* < .05). Specifically, levels of retinol and γ-tocopherol were higher in the MetS group. In contrast, concentrations of 6 carotenoids were lower in the MetS group: α-carotene, trans-β-carotene, cis-β-carotene, lutein/zeaxanthin, α-cryptoxanthin, and β-cryptoxanthin.

**Table 4:** Serum levels of fat-soluble antioxidants in U.S. adults with and without metabolic syndrome (MetS) (NHANES 2017–2018).

Vitamin A, vitamin E, and carotenoids	MetS (n = 426)	Non-MetS (n = 207)	*P* value
	Mean ± SD (µg/dL), n (%)	Mean ± SD (µg/dL), n (%)	
Vitamin A			
Retinol	60.94 ± 18.48 (426–67.30%)	56.90 ± 14.89 (207–32.70%)	.008*
Retinyl palmitate	1.71 ± 1.83 (395–67.40%)	1.67 ± 1.43 (191–32.59%)	.343
Retinyl stearate	0.66 ± 0.55 (386–67.01%)	0.59 ± 0.28 (190–33.00%)	.345
Vitamin E			
γ-Tocopherol	165.79 ± 104.98 (420–67.00%)	145.79 ± 75.07 (207–33.01%)	.034*
α-Tocopherol	1460.82 ± 604.65 (420–67.42%)	1358.59 ± 468.09 (203–32.58%)	.115
Carotenoids			
α-Carotene	4.12 ± 4.51 (397–67.17%)	7.10 ± 17.69 (194–32.83%)	.009*
Trans-β-carotene	22.49 ± 22.9 (409–66.94%)	29.55 ± 41.50 (202–33.06%)	.023*
Cis-β-carotene	1.27 ± 1.21 (389–67.65%)	1.69 ± 2.32 (186–32.35%)	.011*
Lutein/zeaxanthin	21.17 ± 17.39 (400–66.78%)	24.87 ± 23.88 (199–33.22%)	.020*
Trans-lycopene	16.33 ± 9.00 (409–67.38%)	17.26 ± 10.26 (198–32.62%)	.475
Total lycopene	30.19 ± 16.08 (403–67.28%)	33.50 ± 19.43 (196–32.72%)	.092
α-Cryptoxanthin	2.2 ± 1.34 (408–67.00%)	2.51 ± 1.51 (201–33.00%)	.009*
β-Cryptoxanthin	8.61 ± 11.9 (406–66.56%)	8.74 ± 6.96 (204–33.44%)	.005*

* *P* < .05.

**Figure 2.**
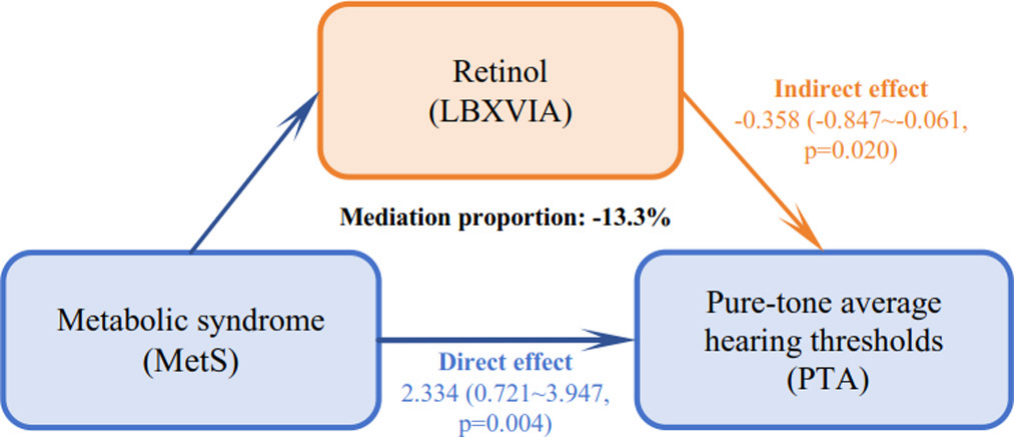
Mediation analysis of serum retinol (LBXVIA) on the association between metabolic syndrome (MetS) and pure-tone average (PTA) hearing thresholds in U.S. adults from NHANES 2017 to 2018. This mediation analysis was conducted using weighted generalized linear models with nonparametric bootstrap resampling (5000 simulations) to account for the complex sampling design of the National Health and Nutrition Examination Survey (NHANES), including survey weights, stratification, and primary sampling units. The indirect effect of MetS on PTA via serum retinol (LBXVIA) was −0.358 (95% CI: −0.847 to −0.061; *P* = .020), accounting for a mediation proportion of −13.3% and indicating an inhibitory pathway where retinol counteracts the adverse effect of MetS on hearing thresholds. The direct effect of MetS on PTA, independent of serum retinol (LBXVIA), remained positive and significant (2.334, 95% CI: 0.721–3.947; *P* = .004). All models were adjusted for age, sex, race, education, marital status, family poverty–income ratio, noise exposure, and history of ear infections. CI, confidence interval; LBXVIA, serum retinol measurement variable; MetS, metabolic syndrome; PTA, pure-tone average hearing threshold; NHANES, National Health and Nutrition Examination Survey.

Causal mediation analysis was performed to evaluate the mediating roles of the 13 fat-soluble antioxidants in the MetS–PTA association, with detailed results presented in Table 5 and the mediation pathway of retinol shown in Figure 2. Only retinol exhibited a statistically significant indirect effect (β = −0.358, 95% CI: −0.847 to −0.061, *P* = .020), with a mediation proportion of −13.3%. The direct effect of MetS on PTA (independent of retinol) was statistically significant and positive (β = 2.334, 95% CI: 0.721–3.947, *P* = .004). The indirect mediating effects of the other 12 fat-soluble antioxidants were not statistically significant (all *P* > .05).

**Table 5:** Mediation analysis of fat-soluble antioxidants in the association between metabolic syndrome (MetS) and pure-tone average (PTA) hearing thresholds (NHANES 2017–2018).

	Indirect effect		Direct effect		
	β (95% CI)	*P* value	Direct effect	*P* value	Mediation
Vitamin A					
Retinol	−0.358 (−0.847 to −0.061)	.020*	3.049 (2.642 to 6.923)	<.001***	−0.133
Retinyl palmitate	−0.127 (−0.22 to 0.267)	.756	2.351 (−2.857 to 4.058)	.644	−0.057
Retinyl stearate	−0.226 (−0.547 to 0.002)	.056	2.581 (−1.467 to 4.834),	.316	−0.096
Vitamin E					
γ-Tocopherol	−0.186 (−0.429 to 0.135)	.468	2.662 (1.009 to 6.675)	.012*	−0.075
α-Tocopherol	−0.126 (−0.292 to 0.167)	.728	2.911 (2.503 to 8.149)	<.001***	−0.045
Carotenoids					
α-Carotene	−0.113 (−0.139 to 0.17)	.752	2.938 (−0.047 to 5.946)	.056	−0.040
Trans-β-carotene	0.064 (−0.085 to 0.178)	.804	2.615 (0.342 to 6.072)	.016*	0.024
Cis-β-carotene	−0.011 (−0.193 to 0.202)	.968	2.450 (−1.651 to 5.181)	.252	−0.004
Lutein/zeaxanthin	0.085 (−0.256 to 0.124)	.620	2.196 (1.234 to 7.367)	.004**	0.037
Trans-lycopene	0.142 (−0.091 to 0.861)	.140	2.594 (−2.697 to 2.834)	.964	0.052
Total lycopene	0.228 (−0.257 to 0.569)	.644	2.622 (−2.546 to 3.815)	.680	0.080
α-Cryptoxanthin	0.167 (−0.117 to 0.411)	.512	2.552 (−2.239 to 3.625)	.596	0.061
β-Cryptoxanthin	0.013 (−0.127 to 0.376)	.632	2.769 (−2.077 to 4.487)	.512	0.005

* *P* < .05.

** *P* < .01.

*** *P* < .001.

## 4. Discussion

This study utilized data from the NHANES to investigate the association between MetS and hearing thresholds among U.S. adults, to assess potential heterogeneity in this relationship across population subgroups, and to evaluate the mediating roles of fat‑soluble antioxidants – specifically vitamins A, E, and carotenoids – in order to provide observational evidence for shared pathological mechanisms underlying these conditions. Key findings were as follows: (1) MetS was independently associated with elevated PTA thresholds after comprehensive covariate adjustment in this national adult sample; (2) subgroup analyses indicated that the association was significantly stronger among older adults (≥50 years), non-Hispanic Whites, middle-income individuals, and those with a history of ear infections; (3) among 13 fat-soluble antioxidants examined, only serum retinol (vitamin A) demonstrated a significant mediating effect.

Our final analytical sample was defined by the availability of complete data for the core variables. Comparisons between included and excluded participants showed minimal differences in most baseline characteristics. Although a modest disparity in education level was observed (SMD = –0.16), it is unlikely to introduce substantial bias, confirming that the data completeness requirement did not lead to significant selection bias. Therefore, the final sample remains broadly representative of the target NHANES adult population, supporting the internal validity of our findings and strengthening the inferential basis of this study.

This study delineates the epidemiological overlap between MetS and hearing loss in U.S. adults. The weighted prevalence of MetS was 35.24%, and the prevalence of hearing loss (PTA ≥ 20 dB) was 22.66% in the overall sample, with a markedly higher rate observed among individuals with MetS (37.12% vs 14.79%) (Section 3.3). This pattern of strong association is corroborated by large-scale studies in other ethnic populations. In a study of 94,223 Korean adults, the average hearing thresholds were significantly higher in individuals with MetS across all age groups, and the prevalence of hearing loss was significantly greater in those with MetS during their 30s to 50s.^[33]^ Similarly, a cross-sectional study of 18,824 middle-aged and older Chinese adults reported a hearing loss prevalence of 52.3%, which was significantly higher in those with MetS (54.9% vs 50.7%).^[25]^The profiles of participants with each condition revealed important and partially distinct patterns: those with MetS were characteristically older and more likely to be female (Table S3, Supplemental Digital Content 3), whereas participants with hearing loss were older and had a higher proportion of males (Table 1). Both groups shared characteristics such as a lower prevalence of higher education. These distributions confirm that key demographic and socioeconomic factors are associated with both conditions. Crucially, to ensure that the estimated association between MetS and hearing thresholds was not confounded by these underlying participant characteristics, all primary multivariable and subgroup analyses were rigorously adjusted for age, sex, race, education, and income to isolate the independent association of MetS.

Our finding of a significant, independent association between MetS and elevated hearing thresholds aligns with and extends a growing body of evidence from diverse populations. Previous studies, including large health-screening cohorts in Korea (n = 94,223), occupational cohorts of professional drivers in Iran (n = 11,114), and community-based samples of middle-aged and older adults in China (n = 18,824), have consistently reported positive associations between MetS or its components and hearing loss.^[24–26]^ However, many of these studies were conducted in specific demographic subgroups (e.g., older adults, occupational drivers) or within single national contexts, which may introduce selection bias and limit generalizability. Moreover, not all prior analyses fully accounted for key hearing-related confounders such as detailed noise exposure history and prior ear infections. The present analysis extends this evidence by using a large, nationally representative sample of U.S. adults from NHANES, with complex survey weighting to ensure population representativeness. Importantly, our models adjusted for a comprehensive set of potential confounders – including socioeconomic factors, noise exposure, and history of ear infections – that were not uniformly available or controlled for in earlier studies. This rigorous methodological approach reduces the selection bias and residual confounding often present in studies of restricted or occupational populations, thereby providing a more accurate and generalizable estimate of the MetS–hearing loss association for the general U.S. adult population.

Although the effect size of MetS on hearing thresholds was modest (β = 1.292 dB), its statistical significance translates to meaningful public health and clinical relevance given the high population prevalence of MetS (35.24% in our cohort). Importantly, our cross-sectional design may underestimate the long-term impact of chronic metabolic dysregulation on auditory function, as this association is likely a gradual, cumulative process spanning decades. Furthermore, even small, incremental elevations in PTA can contribute to the development of mild hearing loss – a clinically underrecognized precursor to severe auditory impairment that is linked to social disengagement, cognitive decline, and reduced quality of life. This modest effect size aligns with findings from other large-scale studies. In a study of 11,114 Iranian adults, MetS was associated with significantly higher odds of hearing loss (OR, 1.38; 95% CI: 1.25–1.51).^[20]^ Similarly, a study of 18,824 Chinese adults reported a positive association (OR, 1.11; 95% CI: 1.03–1.19).^[21]^ The consistency of these modest but significant associations across diverse populations supports the robustness of the MetS–hearing loss relationship and reinforces its potential as a modifiable target for prevention strategies.

Through systematic subgroup interaction analyses, this work reveals, for the first time, population heterogeneity in the impact of MetS on hearing. Notably, the association between MetS and hearing loss was significantly stronger in individuals aged ≥50 years (interaction *P* = .008), a finding that may reflect cumulative cochlear microvascular damage caused by long-term metabolic abnormalities.^[34]^ The finding of a stronger MetS–hearing loss association in non-Hispanic White participants extends previous national observations indicating that this group retains a higher risk of hearing impairment independent of noise exposure, suggesting potential intrinsic contributions.^[35]^ While the underlying mechanisms remain to be fully elucidated, 1 plausible hypothesis involves population-specific differences in the frequency of genetic variants associated with auditory function. For instance, certain pathogenic variants in deafness-related genes such as GJB2, MYO7A, and SLC26A4 have been reported to exhibit higher frequencies in non-Hispanic White populations,^[36]^ which could potentially contribute to the observed racial disparity. However, given the lack of genetic data in the current study, this hypothesis requires further investigation in future research incorporating genetic analyses. Additionally, dietary factors may play a role; diets high in ultra-processed foods have been linked to hearing loss.^[37]^ The weaker association observed in minority groups might reflect additional protective mechanisms. One compelling hypothesis is that greater cochlear melanin content in darker-skinned individuals could provide enhanced antioxidant and metal-chelating capacity, offering resilience against oxidative stress from both ultra-processed foods and MetS. Nevertheless, these proposed mechanisms remain speculative and warrant direct examination in future studies. Socioeconomically, the association between MetS and hearing loss is significantly stronger in middle-to-high income groups. This may be attributed to greater occupational stress and sedentary behaviors within this demographic, coupled with insufficient attention to early-stage hearing impairment. Although MetS is more readily diagnosed in this population, suboptimal long-term management may further amplify the risk of hearing damage through cumulative microvascular injury to the cochlea. Most importantly, this research is the first to report a synergistic damaging effect of a history of ear infections and MetS on hearing. A hypothesized underlying mechanism is as follows: previous ear infections have already caused organic damage to middle ear sound-conducting structures and inner ear sensory epithelia; meanwhile, MetS further exacerbates pathological changes in the auditory system and reduces its compensatory capacity by promoting systemic chronic low-grade inflammation and impairing local immune defense functions.^[38,39]^ These subgroup findings provide important evidence for the precise identification of high-risk populations.

The robustness of our primary findings is further supported by comprehensive sensitivity analyses. Repeating the main analysis using a complete-case approach that excluded all participants with missing covariates yielded nearly identical effect estimates (Table 3), confirming that missing data did not meaningfully distort the observed association. Furthermore, sensitivity analyses stratified by NHANES survey cycle reinforced the temporal validity of the association. A consistent and statistically significant positive association between MetS and PTA was observed in the 2 larger cycles encompassing adults aged 20 to 69 years (2011–2012 and 2015–2016). Some variation was noted in cycles restricted to older adults (≥70 years). The point estimate was largest in the 2017 to 2018 cycle, though statistical significance was not maintained in fully adjusted models, likely due to the reduced sample size in this age-restricted cohort. No significant association was found in the 2009 to 2010 senior cohort. These discrepancies between the 2 senior-only cycles likely reflect cross-sectional heterogeneity, survival bias, or differences in cohort composition inherent in very old populations. Crucially, the consistent and significant associations demonstrated in the larger, combined 20 to 69-year-old cohorts affirm that the primary MetS–hearing loss link is reproducible across independent survey periods and not an artifact of a single cycle. Together, these methodological checks enhance confidence in the reliability and internal validity of the reported results.

Among the 13 fat-soluble antioxidants examined, only retinol was identified as a significant partial mediator in the association between MetS and hearing loss. The indirect effect of retinol was negative (β = −0.358, 95% CI: −0.847 to −0.061, *P* = .020), accounting for a mediation proportion of −13.3%. In contrast, the direct effect of MetS on PTA, independent of retinol, remained positive and significant (β = 2.334, 95% CI: 0.721–3.947, *P* = .004), resulting in a positive total effect (β = 1.292). This suggests that retinol exerts a protective (threshold-lowering) influence that partially counteracts the deleterious (threshold-elevating) impact of MetS on hearing. However, the net effect of MetS remains detrimental, indicating that the protective capacity of endogenous retinol is insufficient to fully offset the metabolic damage to the auditory system.Interestingly, serum retinol concentrations were higher in the MetS group, a finding that aligns with previous reports.^[40,41]^ This may reflect a compensatory upregulation of retinol in response to the heightened oxidative stress associated with MetS, consistent with the interpretation that retinol is mobilized as an antioxidant defense, albeit one that cannot completely preserve cochlear function. The protective link between retinol and auditory function is supported by external evidence: studies have reported inverse correlations between serum retinol (or dietary vitamin A) levels and the prevalence or severity of hearing loss.^[42–44]^ Mechanistically, inner ear hair cells and vascular systems are highly sensitive to oxidative stress, and MetS can lead to elevated systemic oxidative stress levels.^[38,45]^ As an important endogenous antioxidant, retinol may exert protective effects by scavenging free radicals and inhibiting lipid peroxidation.^[46,47]^ Notably, recent animal experiments have confirmed that retinoic acid, the active metabolite of retinol, can effectively attenuate noise-induced hearing loss, with intratympanic administration showing protective electrophysiological effects following acoustic trauma,^[48]^ which provides important clues for understanding the protective mechanism of retinol. Collectively, our mediation analysis and these consistent observations position retinol as a key modifiable factor at the intersection of metabolism and hearing. Future mechanistic and interventional studies are warranted to validate its clinical significance and explore the therapeutic potential of retinol-pathway modulation for hearing protection.

In conclusion, this study confirms the association between MetS and hearing loss and identifies retinol as a partial mediator. The study’s strengths include its novelty as the first to systematically evaluate retinol as a mediator in the MetS–hearing loss relationship. Methodologically, it leverages a large, nationally representative sample from NHANES. Moreover, weighted analyses ensure that the results are well-representative of the U.S. adult population. Additional methodological strengths include comprehensive adjustment for key demographic, socioeconomic, and hearing-specific confounders, as well as rigorous sensitivity analyses that support the robustness of the findings.

However, this study has some limitations: first, the cross-sectional design cannot determine the causal temporality between MetS, retinol, and hearing loss; prospective cohort studies are ultimately required to establish temporality and causality. Second, the fat-soluble antioxidants levels were measured only once, which may not fully reflect long-term exposure. Meanwhile, the availability of fat-soluble antioxidants data from only 1 cycle may introduce selection bias. To better capture habitual nutritional status and reduce measurement variability, future research should include serial measurements of vitamins and carotenoids, ideally repeated across multiple survey cycles. Third, the causes of hearing loss were not classified, which limits in-depth understanding of the association between MetS and different types of hearing loss. Future studies that incorporate such distinctions would enable a more nuanced understanding of the relationship.

These findings not only deepen the understanding of the mechanism underlying metabolic hearing loss but also provide a scientific basis for developing targeted prevention and intervention strategies. Future research should focus on exploring the potential value of preventing hearing loss through metabolic regulation and retinol-related pathways.

## Author contributions

**Conceptualization:** Weiqun Hu, Huiting Dai, Xiufen Chen.

**Formal analysis:** Zhiyong Pan.

**Investigation:** Linwei Mao.

**Methodology:** Zhiyong Pan.

**Project administration:** Huiting Dai.

**Resources:** Weiqun Hu.

**Software:** Xiufen Chen.

**Validation:** Huiting Dai.

**Writing – original draft:** Linwei Mao.









## References

[1] Chadha S Kamenov K Cieza A. The world report on hearing, 2021. Bull World Health Organ. 2021;99:242–242A. 33953438 10.2471/BLT.21.285643 PMC8085630

[2] Statistics NCFH. Percentage of any difficulty hearing for adults aged 18 and over, United States, 2019—2024. National Health Interview Survey. 2026(2026-1-31)

[3] Danis DOR Jain R Homer BJ O’Brien M Gall EK Noonan KY. Nationwide hearing loss trends over two decades. The Laryngoscope. 2025;135:277–85. 39087526 10.1002/lary.31671

[4] Wei J Li Y Gui X. Association of hearing loss and risk of depression: a systematic review and meta-analysis. Front Neurol. 2024;15:1446262–1446262. 39497727 10.3389/fneur.2024.1446262 PMC11532142

[5] Chakrabarty S Mudar R Chen Y Husain FT. Contribution of tinnitus and hearing loss to depression: NHANES population study. Ear Hear. 2024;45:775–86. 38291574 10.1097/AUD.0000000000001467

[6] Qin A Chen C Bao B Xin T Xu L. Estimating the impact of different types hearing loss on cognitive decline and the joint effect of hearing loss and depression on cognitive decline among older adults in China. J Affect Disord. 2024;351:58–65. 38286235 10.1016/j.jad.2024.01.203

[7] The L. Prioritising prevention of hearing loss. Lancet. 2019;393:848. 10.1016/S0140-6736(19)30403-9 30837130

[8] Zhang L Du Z He L Liang W Liu K Gong S. ROS-induced oxidative damage and mitochondrial dysfunction mediated by inhibition of SIRT3 in cultured cochlear cells. Neural Plast. 2022;2022:5567174. 35096052 10.1155/2022/5567174 PMC8791755

[9] Li P Li S Wang L. Mitochondrial dysfunction in hearing loss: oxidative stress, autophagy and NLRP3 inflammasome. Front Cell Dev Biol. 2023;11:1119773–1119773. 36891515 10.3389/fcell.2023.1119773 PMC9986271

[10] Blaner WS Shmarakov IO Traber MG. Vitamin A and Vitamin E: will the real antioxidant please stand up? Annu Rev Nutr. 2021;41:105–31. 34115520 10.1146/annurev-nutr-082018-124228

[11] Curhan SG Stankovic KM Eavey RD Wang M Stampfer MJ Curhan GC. Carotenoids, vitamin A, vitamin C, vitamin E, and folate and risk of self-reported hearing loss in women. Am J Clin Nutr. 2015;102:1167–75. 26354537 10.3945/ajcn.115.109314 PMC4625586

[12] Paz MFCJ Braga AL de Meneses APM. Ascorbic acid and retinol palmitate modulatory effect on omeprazole-induced oxidative damage, and the cytogenetic changes in S. cerevisiae and S180 cells. Chem Biol Interact. 2019;311:108776. 31369745 10.1016/j.cbi.2019.108776

[13] Vašková J Stupák M Vidová Ugurbaş M Židzik J Mičková H. Therapeutic uses of retinol and retinoid-related antioxidants. Molecules (Basel, Switzerland). 2025;30:2191. 40430363 10.3390/molecules30102191 PMC12114363

[14] Ono K Sandell LL Trainor PA Wu DK. Retinoic acid synthesis and autoregulation mediate zonal patterning of vestibular organs and inner ear morphogenesis. Development (Cambridge, England). 2020;147:dev192070. 32665247 10.1242/dev.192070 PMC7420839

[15] Chakraborty S Wang S Ruhala J Mehling B Liu J Waldhaus J. Retinoic acid receptor assembly dynamics governs dual functions in cochlear organogenesis. Proc Natl Acad Sci U S A. 2025;122:e2426739122–e2426739122. 40577120 10.1073/pnas.2426739122 PMC12232719

[16] Carles L Gibaja A Scheper V. Efficacy and mechanisms of antioxidant compounds and combinations thereof against cisplatin-induced hearing loss in a rat model. Antioxidants (Basel, Switzerland). 2024;13:761. 39061830 10.3390/antiox13070761 PMC11273477

[17] Alvarado JC Fuentes-Santamaria V Melgar-Rojas P Gabaldón-Ull MC Cabanes-Sanchis JJ Juiz JM. Oral antioxidant vitamins and magnesium limit noise-induced hearing loss by promoting sensory hair cell survival: role of antioxidant enzymes and apoptosis genes. Antioxidants (Basel). 2020;9:1177. 33255728 10.3390/antiox9121177 PMC7761130

[18] Darvin ME Lademann J von Hagen J. Carotenoids in human skinin vivo: antioxidant and photo-protectant role against external and internal stressors. Antioxidants (Basel). 2022;11:1451. 35892651 10.3390/antiox11081451 PMC9394334

[19] Fu Y Chen W Guo L Liu Y. The inverted-U relationship between dietary inflammatory potential and hearing loss among adults aged 20 years and over in the United States: a cross-sectional study. J Inflamm Res. 2021;14:6671–83. 34916819 10.2147/JIR.S337737 PMC8669755

[20] Min X Kong X Wang W. L-shaped associations between composite dietary antioxidant index and hearing loss: a cross-sectional study from the national health and nutrition examination survey. Biol Res Nurs. 2025;27:28–36. 38869482 10.1177/10998004241261400

[21] Neeland IJ Lim S Tchernof A. Metabolic syndrome. Nat Rev Dis Primers. 2024;10:77. 39420195 10.1038/s41572-024-00563-5

[22] Abohashem S Hassan I Wasfy JH Taub PR. Trends and prevalence of the metabolic syndrome among US adults. JAMA. 2026;335:274–7. 41379435 10.1001/jama.2025.21712 PMC12699394

[23] Yang C Jia X Wang Y. Trends and influence factors in the prevalence, intervention, and control of metabolic syndrome among US adults, 1999-2018. BMC Geriatr. 2022;22:979–979. 36536296 10.1186/s12877-022-03672-6 PMC9764589

[24] Aghazadeh-Attari J Mansorian B Mirza-Aghazadeh-Attari M Ahmadzadeh J Mohebbi I. Association between metabolic syndrome and sensorineural hearing loss: a cross-sectional study of 11,114 participants. Diabetes Metab Syndr Obes. 2017;10:459–65. 29138586 10.2147/DMSO.S150893 PMC5680967

[25] Han X Wang Z Wang J. Metabolic syndrome is associated with hearing loss among a middle-aged and older Chinese population: a cross-sectional study. Ann Med. 2018;50:587–95. 29693425 10.1080/07853890.2018.1469786

[26] Rim H Kim M Park D. Association of metabolic syndrome with sensorineural hearing loss. J Clin Med. 2021;10:4866. 34768385 10.3390/jcm10214866 PMC8584388

[27] Das D Shruthi NR Banerjee A Jothimani G Duttaroy AK Pathak S. Endothelial dysfunction, platelet hyperactivity, hypertension, and the metabolic syndrome: molecular insights and combating strategies. Front Nutr. 2023;10:1221438–1221438. 37614749 10.3389/fnut.2023.1221438 PMC10442661

[28] Tran V De Silva TM Sobey CG. The vascular consequences of metabolic syndrome: rodent models, endothelial dysfunction, and current therapies. Front Pharmacol. 2020;11:148–148. 32194403 10.3389/fphar.2020.00148 PMC7064630

[29] Romeo S Valenti L. Regulation of retinol-binding protein 4 and retinol metabolism in fatty liver disease. Hepatology (Baltimore, Md.). 2016;64:1414–6. 10.1002/hep.28722 PMC5129561 27396306

[30] Sharebiani H Mokaram M Mirghani M Fazeli B Stanek A. The effects of antioxidant supplementation on the pathologic mechanisms of metabolic syndrome and cardiovascular disease development. Nutrients. 2024;16:1641. 38892574 10.3390/nu16111641 PMC11175159

[31] Goncalves A Amiot M. Fat-soluble micronutrients and metabolic syndrome. Curr Opin Clin Nutr Metab Care. 2017;20:492–7. 28858890 10.1097/MCO.0000000000000412 PMC5639995

[32] Gunanti IR Marks GC Al-Mamun A Long KZ. Low serum concentrations of carotenoids and vitamin E are associated with high adiposity in Mexican-American children. J Nutr. 2014;144:489–95. 24500938 10.3945/jn.113.183137

[33] Han H Zhang S Wang M. Retinol metabolism signaling participates in microbiota-regulated fat deposition in obese mice. J Nutr Biochem. 2025;136:109787–109787. 39461600 10.1016/j.jnutbio.2024.109787

[34] Felicio JS de Souza D’Albuquerque Silva L Martins CLEL. Cochlear dysfunction and microvascular complications in patients with type 1 diabetes mellitus. Diabetol Metab Syndr. 2018;10:81. 30455746 10.1186/s13098-018-0380-z PMC6230237

[35] Hoffman HJ Dobie RA Losonczy KG Themann CL Flamme GA. Declining prevalence of hearing loss in US adults aged 20 to 69 years. JAMA Otolaryngol Head Neck Surg. 2017;143:274–85. 27978564 10.1001/jamaoto.2016.3527 PMC5576493

[36] Peart L Gonzalez J Morel Swols D. Dispersed DNA variants underlie hearing loss in South Florida’s minority population. Hum Genomics. 2023;17:103–103. 37996878 10.1186/s40246-023-00556-7 PMC10668374

[37] Fu Y Chen W Liu Y. The association between ultra-processed food intake and age-related hearing loss: a cross-sectional study. BMC Geriatr. 2024;24:450–450. 38783172 10.1186/s12877-024-04935-0 PMC11118724

[38] Masenga SK Kabwe LS Chakulya M Kirabo A. Mechanisms of oxidative stress in metabolic syndrome. Int J Mol Sci. 2023;24:7898. 37175603 10.3390/ijms24097898 PMC10178199

[39] Wijngaarden LH van der Harst E Klaassen RA. Effects of morbid obesity and metabolic syndrome on the composition of circulating immune subsets. Front Immunol. 2021;12:675018. 34354700 10.3389/fimmu.2021.675018 PMC8330422

[40] Kim T Kang J. Association between serum retinol and alpha-tocopherol levels and metabolic syndrome in Korean general population: analysis of population-based nationally representative data. Nutrients. 2020;12:1689. 32516964 10.3390/nu12061689 PMC7352386

[41] Qorbani M Seif E Heshmat R. Association of serum retinol concentrations with metabolic syndrome components in iranian children and adolescents: the CASPIAN-V study. Front Nutr. 2022;9:807634. 35634391 10.3389/fnut.2022.807634 PMC9137422

[42] Kim TS Chung JW. Associations of dietary riboflavin, niacin, and retinol with age-related hearing loss: an analysis of korean national health and nutrition examination survey data. Nutrients. 2019;11:896. 31010085 10.3390/nu11040896 PMC6520829

[43] Slotkowski R Van Ormer M Akbar A. Retinol and pro-Vitamin A carotenoid nutritional status during pregnancy is associated with newborn hearing screen results. Nutrients. 2023;15:800. 36839158 10.3390/nu15040800 PMC9967333

[44] Lee HJ Lee J Yoon C. Association of dietary factors with noise-induced hearing loss in Korean population: a 3-year national cohort study. PLoS One. 2022;17:e0279884–e0279884. 36584228 10.1371/journal.pone.0279884 PMC9803270

[45] Teraoka M Hato N Inufusa H You F. Role of oxidative stress in sensorineural hearing loss. Int J Mol Sci . 2024;25:4146. 38673731 10.3390/ijms25084146 PMC11050000

[46] Didier AJ Stiene J Fang L Watkins D Dworkin LD Creeden JF. Antioxidant and anti-tumor effects of dietary vitamins A, C, and E. Antioxidants (Basel). 2023;12:632. 36978880 10.3390/antiox12030632 PMC10045152

[47] Tschuck J Padmanabhan Nair V Galhoz A. Suppression of ferroptosis by vitamin A or radical-trapping antioxidants is essential for neuronal development. Nat Commun. 2024;15:7611. 39218970 10.1038/s41467-024-51996-1 PMC11366759

[48] Uysal GS Erbek S Erbek HS Özlüoğlu LN. Electrophysiological effects of intratympanic retinoic acid application following acoustic trauma in rats. J Int Adv Otol. 2025;21:1–6. 10.5152/iao.2025.241834 PMC12128312 40522129

