# Supplementary material for: Association between metabolic syndrome and hearing loss: The mediating role of retinol – A cross-sectional analysis of NHANES 2007 to 2018 (excluding 2013–2014)
Source: Medicine (Baltimore). 2026 Jun 5;105(23):e49234. doi: 10.1097/MD.0000000000049234 (PMC13246054; doi:10.1097/MD.0000000000049234)
Supplement: Supplementary file 1 [file medi-105-e49234-s001.docx]

****Supplementary Table S1.** Comparison of Baseline Characteristics Between Included and Excluded U.S. Adult Participants Aged ≥20 Years (NHANES 2009–2018, Excluding 2013–2014)**

| **Characteristic** | **Included Participants (n=8,759)** | **Excluded Participants (n=19,065)** | SMD |
| --- | --- | --- | --- |
| Age, Mean ± SD | 49.16 ± 17.73 | 50.29 ± 17.81 | -0.06 |
| Sex, n (%) |  |  |  |
| Female | 4477 (51.11) | 9836 (51.59) | -0.01 |
| Male | 4282 (48.88) | 9229 (48.41) |  |
| Race, n (%) |  |  |  |
| Non-Hispanic White | 3259 (37.21) | 7868 (41.27) | -0.08 |
| Non-Hispanic Black | 2017 (23.03) | 4068 (21.34) | 0.04 |
| Others | 3483 (39.76) | 7129 (37.39) | 0.05 |
| Others (Mexican American) | 1200 (13.70) | 3053 (16.01) | -0.07 |
| Others (Other Hispanic) | 954 (10.89) | 2057 (10.79) | 0.00 |
| Others (Other Race) | 1329 (15.17) | 2019 (10.59) | 0.14 |
| Education level, n (%) |  |  |  |
| High school graduate or less | 3783 (43.19) | 9686 (50.90) | -0.15 |
| College or above | 4976 (56.81) | 9343 (49.10) |  |
| Marital status, n (%) |  |  |  |
| Married/living with a partner | 5185 (59.20) | 11218 (58.88) | 0.01 |
| Never married | 1732 (19.77) | 3376 (17.72) | 0.05 |
| Divorced/separated/widowed | 1842 (21.03) | 4458 (23.40) | -0.06 |
| Family PIR, N (%) |  |  |  |
| ≤ 1.3 | 2750 (31.4) | 5628 (33.22) | -0.04 |
| 1.3–3.5 | 3357 (38.33) | 6434 (37.98) | 0.01 |
| > 3.5 | 2652 (30.28) | 4878 (28.80) | 0.03 |

Abbreviations: PIR, Poverty-Income Ratio; SMD, Standardized Mean Difference.

Values are presented with two decimal places. An absolute SMD < 0.10 is generally considered to indicate negligible imbalance between groups.
