# Supplementary material for: Association between metabolic syndrome and hearing loss: The mediating role of retinol – A cross-sectional analysis of NHANES 2007 to 2018 (excluding 2013–2014)
Source: Medicine (Baltimore). 2026 Jun 5;105(23):e49234. doi: 10.1097/MD.0000000000049234 (PMC13246054; doi:10.1097/MD.0000000000049234)
Supplement: Supplementary file 2 [file medi-105-e49234-s002.docx]

**Supplementary Table S2.** Prevalence of Individual Metabolic Syndrome (MetS) Components in the U.S. Adult Study Population (NHANES 2009–2018, Excluding 2013–2014; N=8,759)

| **Metabolic Syndrome Component** | **Overall (n=8,759)** | **Non-MetS (n=5,672)** | **MetS (n=3,087)** | **p-value** |
| --- | --- | --- | --- | --- |
| central obesity | 5779(65.98) | 2929(51.64) | 2850(92.32) | <0.001 |
| hypertriglyceridemia | 3410(38.93) | 1115(19.66) | 2295(74.34) | <0.001 |
| low high-density lipoprotein cholesterol (HDL-C) | 4073(46.5) | 1483(26.15) | 2590(83.9) | <0.001 |
| hypertension | 3889(44.4) | 1276(22.5) | 2613(84.65) | <0.001 |
| hyperglycemia | 3091(35.29) | 842(14.84) | 2249(72.85) | <0.001 |

MetS was defined according to the National Cholesterol Education Program Adult Treatment Panel III (NCEP ATP III).
