# Supplementary material for: Association between metabolic syndrome and hearing loss: The mediating role of retinol – A cross-sectional analysis of NHANES 2007 to 2018 (excluding 2013–2014)
Source: Medicine (Baltimore). 2026 Jun 5;105(23):e49234. doi: 10.1097/MD.0000000000049234 (PMC13246054; doi:10.1097/MD.0000000000049234)
Supplement: Supplementary file 3 [file medi-105-e49234-s003.docx]

| **Total** | All (n  =  8,759) | **MetS (n = 3,087)** | **Non-MetS (n = 5,672)** | P value |
| --- | --- | --- | --- | --- |
| **Sociodemographic Characteristics** |  |  |  |  |
| Age, Mean ± SD | 49.16 ± 17.73 | 59.42 ± 14.65 | 42.57 ± 16.72 | <0.001 |
| Sex, n (%) |  |  |  | <0.001 |
| Female | 4477 (51.11) | 1754 (56.82) | 2723 (48.01) |  |
| Male | 4282 (48.88) | 1333 (43.18) | 2949 (51.99) |  |
| Race, n (%) |  |  |  | <0.001 |
| Non-Hispanic White | 3259 (37.21) | 1243 (40.27) | 2016 (35.54) |  |
| Non-Hispanic Black | 2017 (23.03) | 752 (24.36) | 1265 (22.30) |  |
| Others | 3483 (39.76) | 1092 (35.37) | 2391 (42.15) |  |
| Others (Mexican American) | 1200 (13.70) | 435 (14.09) | 765 (13.49) |  |
| Others (Other Hispanic) | 954 (10.89) | 348 (11.27) | 606 (10.68) |  |
| Others (Other Race) | 1329 (15.17) | 309 (10.01) | 1020 (17.98) |  |
| Education level, n (%) |  |  |  | <0.001 |
| High school graduate or less | 3783 (43.19) | 1533 (49.66) | 2250 (39.67) |  |
| College or above | 4976 (56.81) | 1554 (50.34) | 3422 (60.33) |  |
| Marital status, n (%) |  |  |  | <0.001 |
| Married/living with a partner | 5185 (59.20) | 1813 (58.73) | 3372 (59.45) |  |
| Never married | 1732 (19.77) | 333 (10.79) | 1399 (24.67) |  |
| Divorced/separated/widowed | 1842 (21.03) | 941 (30.48) | 901 (15.88) |  |
| Family PIR, n (%) |  |  |  | <0.001 |
| ≤ 1.3 | 2750 (31.4) | 997 (32.30) | 1753 (30.91) |  |
| 1.3–3.5 | 3357 (38.33) | 1265 (40.98) | 2092 (36.88) |  |
| > 3.5 | 2652 (30.28) | 825 (26.72) | 1827 (32.21) |  |
| **Hearing-Related Risk Factors** |  |  |  |  |
| Noise exposure, n (%) |  |  |  | 0.037 |
| Yes | 4282 (48.90) | 1556 (50.44) | 2726 (48.08) |  |
| No | 4475 (51.10) | 1530 (49.56) | 2945 (51.92) |  |
| History of ear infections, n (%) |  |  |  | <0.001 |
| Yes | 1933 (25.48) | 691 (30.52) | 1242 (21.90) |  |
| No | 5653 (74.52) | 1,694 (74.83) | 3959 (69.80) |  |
| **Hearing loss, n (%)** |  |  |  | <0.001 |
| Yes | 1985 (22.66) | 1146 (37.12) | 839 (14.79) |  |
| No | 6774 (77.34) | 1941 (62.88) | 4833 (85.21) |  |

**Supplementary Table S3.** Baseline Characteristics of the U.S. Adult Study Population (NHANES 2009–2018, Excluding 2013–2014), Stratified by Metabolic Syndrome (MetS) Status

Abbreviation: PIR, Poverty-Income Ratio
