# Supplementary material for: Association between metabolic syndrome and hearing loss: The mediating role of retinol – A cross-sectional analysis of NHANES 2007 to 2018 (excluding 2013–2014)
Source: Medicine (Baltimore). 2026 Jun 5;105(23):e49234. doi: 10.1097/MD.0000000000049234 (PMC13246054; doi:10.1097/MD.0000000000049234)
Supplement: Supplementary file 4 [file medi-105-e49234-s004.docx]

**Supplementary Table S4.** Sensitivity Analysis of the Association Between Metabolic Syndrome (MetS) and Pure-Tone Average (PTA) Hearing Thresholds, Stratified by NHANES Survey Cycle (2009–2018, Excluding 2013–2014).

| **Survey Cycle** | **Age Range** | ***Sample Size (n)*** | **Model 1 (demographic-adjusted)** | | **Model 2 (socioeconomic-adjusted)** | | **Model 3**  **(fully-adjusted)** | |
| --- | --- | --- | --- | --- | --- | --- | --- | --- |
|  |  |  | **β (95%CI)** | **P value** | **β (95%CI)** | **P value** | **β (95%CI)** | **P value** |
| 2009-2010 | ≥70 years | 718 | -0.93 (-3.81, 1.95) | 0.536 | -1.28(-4.25, 1.69) | 0.419 | -1.30 -4.25,1.65) | 0.416 |
| 2011-2012 | 20-69 years | 3,518 | 1.51 (0.53, 2.48) | 0.010 | 1.29 (0.36, 2.23) | 0.022 | 1.18 (0.24, 2.12) | 0.039 |
| 2015-2016 | 20-69 years | 3,806 | 2.77 (1.93, 3.61) | 0.001 | 2.51 (1.72, 3.29) | 0.001 | 2.28 (1.52, 3.04) | 0.001 |
| 2017-2018 | ≥70 years | 654 | 3.17 (0.39, 5.95) | 0.047 | 3.10 (0.39, 5.80) | 0.055 | 3.19 (0.50, 5.88) | 0.059 |

Model 1: Adjusted for age, sex, and race.

Model 2: Combination of model 1 and education level, marital status, and family Poverty-Income Ratio (PIR).

Model 3: Combination of model 2 and noise exposure and history of ear infections.
